# 

**Published:** 1916-03-25

**Authors:** 


### CONTENTS.

PAGE

**The Claim of the Doctor** ... ... ... ... ... 559

**Praise and Blame** ... ... ... ... ... ... 560

**Hospital and Institutional News** ... ... 561

Macmillan and Co., Ltd., and Another *v.* The Nursing Press, Ltd., and Others—"Another Brownlow Hill Infirmary Scandal"—Lord Knutsford at Home Again—The Director-General of the A.M.S.—The Conscientious Objector in Military Hospitals—The Pensions of Tuberculous Ex-Soldiers—The late Sir Charles Ball—Hospital Arrangements in Mesopotamia—Guy's Hospital—Doctors Reproved by Insurance Committee—The Hospital Lesson of Bristol—An Irish Philanthropist—Amusement as the Basis of Orderly Conduct—A Desperate Expedient at York—Ordinary Competence and Skill—Prophylaxis no Treatment—The End of an Old Dispensary—The Test of General Ability—The Asylum and Signs of Change—Croydon Hospital without Resident Doctor—The London Mental Hospitals Committee—The *Vis Inertia* in a Scotch Asylum—The New Aberavon Hospital—A Curious Cottage Hospital Practice—Why do Sanatorium Patients Complain?—The Heel of Achilles in Sanatorium Treatment—A Corporation Care Committee—An Educational Campaign to Combat Venereal Disease—This Week's Drug Market.

**A Medical Causerie** ... ... ... ... ... ... 567

**Carbolic-Acid Poisoning** ... ... ... ... ... 568

An Experimental Investigation.

**The War Office and the Poor-Law Infirmary** 569

Military and Civilian Standards.

PAGE

**Research and Remedies** ... ... ... ... ... 570

**The War's Effect on a Special Hospital** ... 571

Phenomenal Increase in the Cost of Treatment.

**Legal Notes for Institutional Workers** ... 572

**The School Doctor** ... ... ... ... ... ... 573

Twenty Years' Experience and Results.

**Schools of Massage** ... ... ... ... ... ... 574

II. St. Thomas's Hospital Physical Exercises and Massage School.

**The Matrons' and Sisters' Department** ... 575

Linen Leagues for Hospitals: Their Growing Activities and Value.
Round the Hospitals.
Medical Staffs and Matrons—Sisters as Anæsthetists—V.A.D.s and Journalism—A Great War Album—Sisters and Smiles.

**The Unconscious Mental Life of the Child** ... 577

Platform of Child Study Society.

**Parliament and the Profession** ... ... ... 577

**The Editor's Letter-Box** ... ... ... ... 578

**Patriotism and the Nurse-Training Schools** ... 579

VII. A Typical Instance of Good Organisation.

**Hospital Results in 1915** ... ... ... ... ... 580

Some Early Reports of the Voluntary Hospitals.

**Hospital Architecture and Construction** ... 584

Empire Hospital for Paying Patients, Vincent Square, S.W.

**The Institutional Worker** ... ... ... (Suppl.) 1

Hospital Inventions.

